# Risk Assessment and Epidemiologic Evidence: Kundi Responds

**Published:** 2006-11

**Authors:** Michael Kundi

**Affiliations:** Institute of Environmental Health, Center for Public Health, Medical University of Vienna, Vienna, Austria, E-mail: Michael.Kundi@meduniwien.ac.at

I appreciate Goldstein’s remarks about the role of epidemiology in risk assessment of environmental hazards and the opportunity to clarify my standpoint.

With reference to the International Agency for Research on Cancer’s classification scheme of agents for their carcinogenicity in humans and other schemes such as that of the U.S. Environmental Protection Agency (EPA), Pitot and Dragan (2001) stated in *Casarett and Doull’s Toxicology*:

In spite of the limitations of these classifications, an agent cannot be proven to be carcinogenic for the human unless substantial epidemiologic evidence supporting such a claim is available.

Although this statement refers to carcinoma and not to the broader class of chronic diseases, it seems to be very close to my statement (Kundi 2006) that Goldstein criticizes. However, Goldstein particularly emphasizes that I may have meant that “there must be epidemiologic evidence for a chemical to achieve the level of a known or proven cause of a hazard to human health.”

The reader may have noticed that I never used the term “proven” (Kundi 2006), and I deliberately did not. In my opinion we cannot reach the level of a proven cause. Our knowledge is always incomplete; although we may be quite sure about a factor causing a disease, it may turn out to be actually unrelated. Using toxicologic evidence, we may conjecture that an agent has a potential to cause human chronic disease, but we need further evidence—in most cases epidemiologic evidence—to establish a causal relationship between the agent and a chronic disease in humans. (I make a conceptual difference between “establishing” and “proving,” the latter defined as “establishing truth,” which can only be done for analytical statements.)

My statement that Goldstein criticizes was misleading insofar as it seems to indicate that we have to start from epidemiologic evidence to ascribe an agent a hazardous potential for human health. In many cases first information on a potential hazard will stem from routine toxicologic testing. The last paragraphs of my commentary (Kundi 2006) were intended to give an outlook to future developments that may provide answers to the question of causation of chronic diseases in a more rapid fashion. From this context it should be clear that risk assessment was addressed with respect to the causal role of an agent. Therefore, a slight modification of the statement above is appropriate: An agent cannot be established to cause a chronic human disease unless supporting epidemiologic evidence is available. Among other improvements, comprehensive utilization of modern molecular biological methods integrated into epidemiologic designs may provide such evidence at an early stage of the disease.
